# Exploring Inflammatory Bowel Disease Discourse on Reddit Throughout the COVID-19 Pandemic Using OpenAI’s GPT-3.5 Turbo Model: Classification Model Validation and Case Study

**DOI:** 10.2196/53332

**Published:** 2025-07-03

**Authors:** Tyler Babinski, Sara Karley, Marita Cooper, Salma Shaik, Y Ken Wang

**Affiliations:** 1 Division of Gastroenterology, Hepatology, and Nutrition Children's Hospital of Philadelphia Philadelphia, PA United States; 2 Division of Management and Education University of Pittsburgh at Bradford Bradford, PA United States; 3 Division of Gastroenterology and Hepatology University of Pennsylvania Philadelphia, PA United States; 4 Department of Child and Adolescent Psychiatry and Behavioral Sciences Children's Hospital of Philadelphia Philadelphia, PA United States

**Keywords:** inflammatory bowel disease, large language model, OpenAI GPT 3.5 Turbo, Reddit, sentiment analysis, topic analysis, ChatGPT, COVID-19, social media, autoimmune disorder, gastrointestinal, machine learning, COVID-19 pandemic, Reddit discourse, classification model, case study

## Abstract

**Background:**

Inflammatory bowel disease (IBD) is a chronic autoimmune disorder with an increasing prevalence in the general population. Internet-based communities have become vital for communication among patients with IBD, especially throughout the COVID-19 pandemic. However, these internet-based patient-to-patient communications remain largely underexplored.

**Objective:**

This study aims to analyze community posts from 3 of the largest IBD support groups on Reddit between March 1, 2020, and December 31, 2022, using a pretrained transformer model, and to validate the classification system’s results via comparison to human scoring.

**Methods:**

We collected posts (N=53,333) from subreddits r/CrohnsDisease, r/UlcerativeColitis, and r/IBD and classified them using OpenAI’s GPT-3.5 Turbo model to determine sentiment, categorize topics, and identify demographic information and mentions of the COVID-19 pandemic. A subset of posts (n=397) was manually scored to measure interrater agreement between human raters and the GPT-3.5 Turbo model.

**Results:**

Fleiss κ and Gwet AC1 coefficients indicated a high level of agreement between raters, with values ranging from 0.53 to 0.91. The raters demonstrated almost perfect agreement on the classification of gender, with a Fleiss κ of 0.91 (*P*<.001). Medications (14,909/53,333) and symptoms (14,939/53,333) emerged as the most discussed topics, and most posts conveyed a neutral sentiment. While most users did not disclose their age, those who did primarily belonged to the 20-29 years (2392/4828) and 30-39 years (859/4828) age groups. Based on self-reported gender, we identified 1509 men and 1502 women among our IBD Reddit users. When comparing the users on the IBD subreddits to the general IBD population, there was a significant difference in gender distribution (N=3,090,011; *χ*^2^_2_=69.53; *P*<.001; φ<0.001). After an initial spike in posts within the first month, most posts did not reference the COVID-19 pandemic.

**Conclusions:**

Our study showcases the potential of generative pretrained transformer models in processing and extracting insights from medical social media data. Future research can benefit from further subanalyses of our validated dataset or use OpenAI’s model to analyze social media data for other conditions, particularly those for which patient experiences are challenging to collect.

## Introduction

Inflammatory bowel disease (IBD) is an autoimmune disorder of the gastrointestinal tract that impacts around 3.1 million adults in the United States [1]. While immunosuppressive medications have shown efficacy in treating IBD, they also increase the risk of infections such as COVID-19 [2,3]. This increased susceptibility to COVID-19 has led individuals with IBD to isolate, potentially exacerbating the adverse health effects associated with pandemic restrictions [4-6]. Despite a substantial body of literature on the use of social media by individuals with IBD, the impact of the COVID-19 pandemic on internet-based discussions within this community remains unclear. Understanding and categorizing behaviors of individuals with IBD can provide insights into how their interactions with social media platforms affect their mental health and inform the development of tailored internet-based resources and support.

Previous studies examining social media use among individuals with IBD have aimed to analyze patient conversations on platforms such as Twitter (subsequently rebranded X) and Reddit (Advance Publications). A 2023 study by Rubin et al [7] examined patient perspectives on factors contributing to ulcerative colitis flares from public forums across 6 countries, identifying >27,000 patient posts, of which (N=12,900, 47.8%) were related to flares. The most frequently reported triggers included stress and anxiety (n=440, 37.9%) and diet (n=330, 28.4%). Another study by Rohde et al [8] characterized topics associated with IBD and distress on Reddit and Twitter, finding that symptoms (n=23,294, 47.8%) and medication (n=12,218, 30.1%) were the most prevalent topics. Additionally, a 2023 study by Stemmer et al [9] analyzed the content and sentiments expressed in posts by patients with IBD, revealing that they expressed more sadness and fear compared with a control group of healthy users. Although this previous research has provided a strong foundation for working with IBD social media data, researchers have encountered difficulties in analyzing the large volumes of posts and validating the findings.

The rapid advancement of machine learning offers a powerful solution to the challenges of analyzing big data. For instance, Goel et al [10] used machine-learning techniques to conduct a sentimental and topical analysis of social media data about endometriosis, another private and stigmatized condition. This study used a bidirectional encoder representation from transformers model, a state-of-the-art natural language processing (NLP) model that can extract insights from the vast amount of unstructured data present in social media discussions. However, training a machine learning model requires substantial funding, computational power, and expertise, limiting the accessibility of this method of data analysis.

GPT-3.5 is a powerful large language model that can generate coherent and diverse texts based on a given input [11]. GPT-3.5 is trained on a large corpus of text from various sources, such as books, websites, news articles, and social media posts. Approximately 22% of its training data came from the OpenWebText corpus, which consists of Reddit posts from 2005 to 2020 [12]. Early data support the use of GPT-3.5 in sentiment and topic analysis, especially within the mental health classification tasks [13-16]. For example, Nadi et al [17] demonstrated support for GPT-3.5 in determining sentiment based on movie reviews, with more than 90% reliability with human coders across multiple datasets. Similarly, He et al [18] compared the performance of GPT-3.5 with the Valence Aware Dictionary for Sentiment Reasoning (VADER) model, an open-source Python package designed to calculate sentiment from free text, finding that GPT-3.5 exhibited greater agreement with human coders in determining sentiment from health-related social media. Despite this, a recent preprint by Lockwood et al [19] highlighted potential flaws in the use of GPT-4 to conduct qualitative coding to identify themes from data by school psychology graduate educators on the impact of COVID-19 on their training, with findings suggesting support for its use in identifying broad themes, but difficulties in elucidating the depth and nuanced interpretation of human coders. However, this study relied on a small sample (N=60), highlighting the need to evaluate the use of NLP in classifying health-related social media data and benchmarking its reliability against human raters.

This study aims to introduce a novel analytical method using GPT-3.5 to analyze large amounts of social media data. Our primary objective is to establish the feasibility of using GPT-3.5 to identify and characterize themes and sentiments in Reddit posts among individuals with IBD during the COVID-19 pandemic. Additionally, we aim to compare the interrater reliability of GPT-3.5 output against human raters to establish the model’s credibility. Finally, this study seeks to contribute to the understanding of discourse among individuals with IBD, particularly during the COVID-19 pandemic.

## Methods

### Data Source and Collection

We collected data from Reddit, a popular social media platform that allows users to create and join communities, or subreddits, based on their interests. Reddit has over 57 million daily users and over 13 billion posts as of 2023 [20]. For this study, data were extracted from the 3 largest subreddits dedicated to IBD: r/CrohnsDisease, r/UlcerativeColitis, and r/IBD. These subreddits serve as internet-based support groups where users can post text, images, videos, or links to other websites and comment on other users’ posts. Each subreddit has its own rules and moderators, who are volunteers overseeing the content and quality of the posts and comments.

We chose to analyze data from March 1, 2020, to December 31, 2022, aligning with the official declaration of the COVID-19 pandemic and its subsequent transition to an endemic phase [21]. We obtained posts from the Pushshift database, an archive of Reddit submissions and comments for researchers [22]. To ensure data integrity, we cross-verified the SHA-256 hash values, a cryptographic hash function designed to confirm data integrity provided by Pushshift, with those we computed for each downloaded file. We used a Python script developed by an open-source contributor to aggregate all subreddit-of-interest submissions into a single Newline Delimited JSON file for each month [23]. These files were subsequently merged into a single CSV file, resulting in an initial dataset of 67,860 posts.

### Data Preprocessing

**Figure 1:**
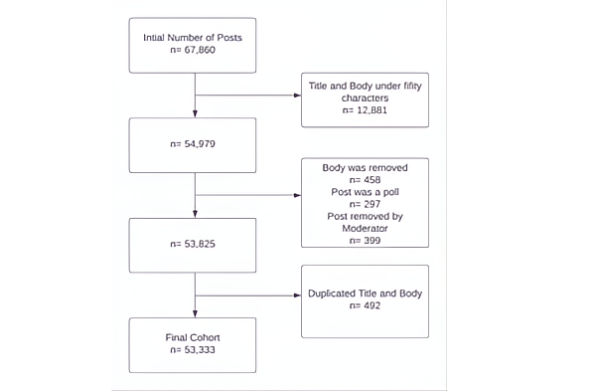
Excluded posts.

We preprocessed the raw data via the following exclusion criteria: combined length ≤50 characters, tagged as a poll, missing a body, posts removed by moderators, and duplicate posts across subreddits. The remaining posts were sorted in ascending order, and each was assigned a unique record ID. The final dataset comprised 53,333 posts. All data cleaning was completed via Alteryx (Alteryx, Inc) [24] (Figure 1).

### Prompt Design and Post Processing

We developed a prompt to evaluate each post’s sentiment with a ternary scale (positive, negative, or neutral) and categorize it into one of 6 areas: medication, treatment, symptoms, diagnosis, diet, or other. Additionally, the prompt identifies any demographic information or references to the COVID-19 pandemic. Since prompt engineering is a relatively new field, we refined the prompt through an iterative process, testing it on random samples from the dataset and adjusting it to validate the stability and accuracy of the sentiment label distributions. The final prompt, shown in Textbox 1, consisted of an initial message that instructed the model about its purpose, followed by instructions for each post-title combination and a final system message that defined the response format. After designing the prompt, we submitted it with each post via a Python script to the GPT-3.5 model application programming interface endpoint in separate batches of 10,000 records to account for website outages and connection losses. We then saved and remerged the responses based on the record ID. The outputs provided by the model were standardized using conditional statements. The recorded ages were grouped into 10-year intervals for demographic analysis.

**Prompt used to classify posts.**

You are a large language model that has been trained to analyze titles and/or bodies of submissions submitted to a Reddit community dedicated to inflammatory bowel disease. The user will submit a list of objectives, and you will respond using only the categories they provide.

“Title and/or Body of post was inserted here”

Determine the sentiment expressed by the user using only the words: Positive, Negative, or Neutral.

Classify the post using one of the following categories: Medication, Treatment, Symptoms, Diagnosis, Diet, or Other.

Extract the gender and age of the poster if they included it in the post. If no demographic information is found, respond with the word 'Null'.

Identify whether the post directly references the COVID-19 pandemic. Report your answer using only the words 'Yes', 'No', or 'Unsure'.

I will only respond in a comma-separated format, as follows:

Sentiment_Goes_Here,Category_Goes_Here,Gender_Goes_Here,Age_Goes_Here,COVID-19_Goes_Here

### Data Validation

To measure the overall accuracy of our model’s classifications, we chose both Fleiss Kappa and Gwet AC1 statistical measures to evaluate interrater reliability. Fleiss Kappa is a widely used statistic for assessing the extent of agreement among multiple raters while accounting for the possibility of chance agreement [25]. Lower Fleiss κ scores (ie, closer to 0) indicate greater disagreement, with scores approaching 1 suggesting higher interrater reliability [26]. We also opted to calculate Gwet AC1 because it is suggested to be less affected by prevalence and marginal probability compared with Fleiss κ, making it a more accurate measure [27]. According to Gwet AC1, scores above 0.75 are deemed acceptable, with higher scores indicating greater agreement.

We calculated the required sample size for this subset analysis using the Taro Yamane Equation with a 0.5 degree of error, which resulted in the selection of 397 posts for evaluation [28-30]. As the sample size for κ coefficients is considered challenging to calculate, this sample size was further cross-referenced against Bujang and Baharum’s [31] prescribed criteria for Cohen κ sample size calculations, confirming an expected sample size of 389 posts. We aimed for an effect size of 0.75. The subsample includes 117 (30%) posts for sentiment evaluation, 49 (12.5%) posts for classification, 71 (18.25%) posts for gender categorization, 35 (9%) posts for age range classification, and an additional 117 (30%) posts for referencing the COVID-19 pandemic.

We generated a randomized set of 397 Reddit posts from the final dataset using Alteryx to ensure impartiality. Two human raters from the study team and GPT-3.5 evaluated each category across multiple predefined categories. To ensure standardization of responses, both human raters followed a predetermined codebook for each category: sentiment (positive, negative, and neutral), category (medication, treatment, symptoms, diagnosis, diet, and other), gender (male and female), age (0-9, 10-19, 20-29, 30-39, 40-49, 50-59, and 60+ years), and reference to COVID-19 (yes, no, and unsure). A small number of posts not included in the subsample were initially reviewed to gather insight. Both human raters reviewed these posts and individually developed definitions for each category. The definitions were then combined to create an established codebook with definitive definitions for each category.

Interrater reliability was assessed by comparing the GPT-3.5 model’s output with the evaluations of the 2 human raters. Any discrepancies identified were returned to the human raters for double scoring independently using the codebook as a reference. The final Fleiss κ and Gwet AC1 analyses were performed using RStudio (R Studio, Inc) and the irrCAC package [32,33].

### Ethical Considerations

The research activities described in this study were reviewed by the Human Research Protection Office at the University of Pittsburgh (STUDY23010103), and the study activities were determined not to involve human subjects as defined by the Department of Health and Human Services (DHHS) and the Food and Drug Administration (FDA) regulations.

## Results

### Data Trends

The comparison between GPT-3.5 and human raters revealed a moderate agreement for sentiment analysis and a substantial concordance for categorization. For variables pertaining to the COVID-19 pandemic references, gender, and age, GPT-3.5 demonstrated almost perfect alignment with human assessments (Table 1).

**Table 1:** Fleiss and Gwet AC1 coefficients for GPT and human raters. All coefficients had a *P* value <.001.

Variables	Fleiss coefficient	Level of agreement	Gwet AC1 coefficient	Level of agreement
Sentiment	0.53	Moderate	0.78	Good
Category	0.69	Substantial	0.72	Good
References COVID-19 pandemic	0.82	Almost perfect	0.98	Very good
Gender	0.91	Almost perfect	0.91	Very good
Age	0.87	Almost perfect	0.91	Very good

**Figure 2:**
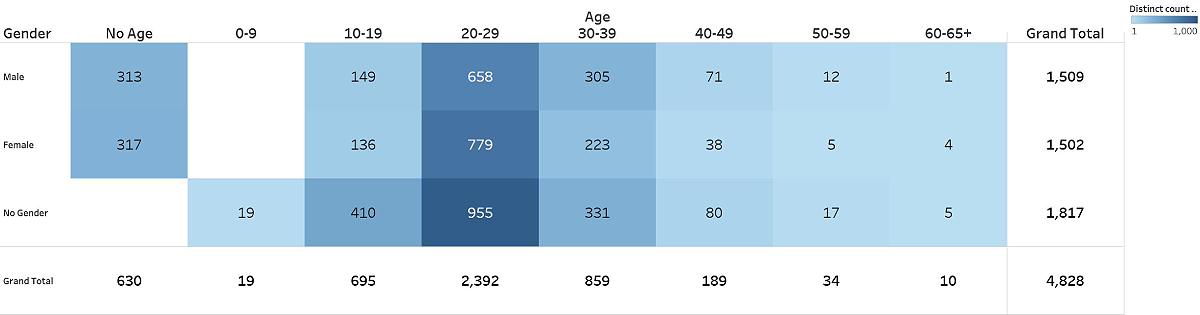
Heatmap of distinct age and gender data.

From self-reported gender, we observed 1509 men and 1502 women in our IBD Reddit users (Figure 2). When comparing the users on the IBD subreddits to the general IBD population, there was a significant difference in gender distribution (N=3,090,011; *χ*^2^_2_=69.53; *P*<.001; φ<0.001). Specifically, we saw a higher proportion of men and fewer women than anticipated considering the overall demographics of those affected by IBD [1]. However, examining the relative effect sizes suggested these differences were negligible. Similarly, while we saw a more significant proportion of women than expected (1144.20; 38%) given the general demographic breakdown of Reddit users (N=50,003,011; *χ*^2^_2_ =180.47; *P*<.001; φ<0.001), our effect size again suggested differences were negligible [34].

Most users posting on the IBD subreddits self-reported their age as between 20-29 years (n=2392, 49%). This was consistent with the results of our chi-square (N=5,000,044; *χ*^2^_4_=1945.51; *P*<.001; Cramer V<0.001), which suggested that users aged between 10-19 and 20-29 years were overrepresented in our IBD Reddit sample, whereas those aged 30-39, 40-49, and 50+ years were underrepresented compared with the general Reddit user data [34]. Again, the investigation of effect sizes suggested these differences were negligible.

**Figure 3:**
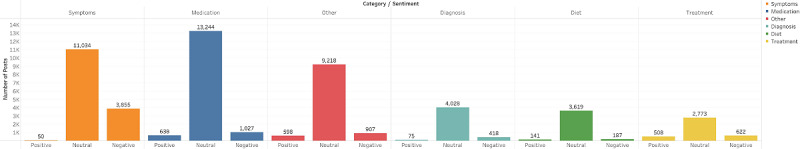
Percentage of posts by category and sentiment.

Sentimental analysis of the posts showed that (n=43,916, 83%) posts were neutral, (n=2010, 4%) were positive, (n=7016, 13%) were negative, and the remaining posts did not have a standardized sentiment value. Comparing this across the topic group (Figure 3) and a previous study, examining topic analysis of Reddit posts discussing IBD exhibited a markedly lower frequency of prepandemic references to diet and nutrition (6204.95). Conversely, there was a notably higher volume of conversations surrounding medications before the pandemic (11,231.93) [8].

**Figure 4:**
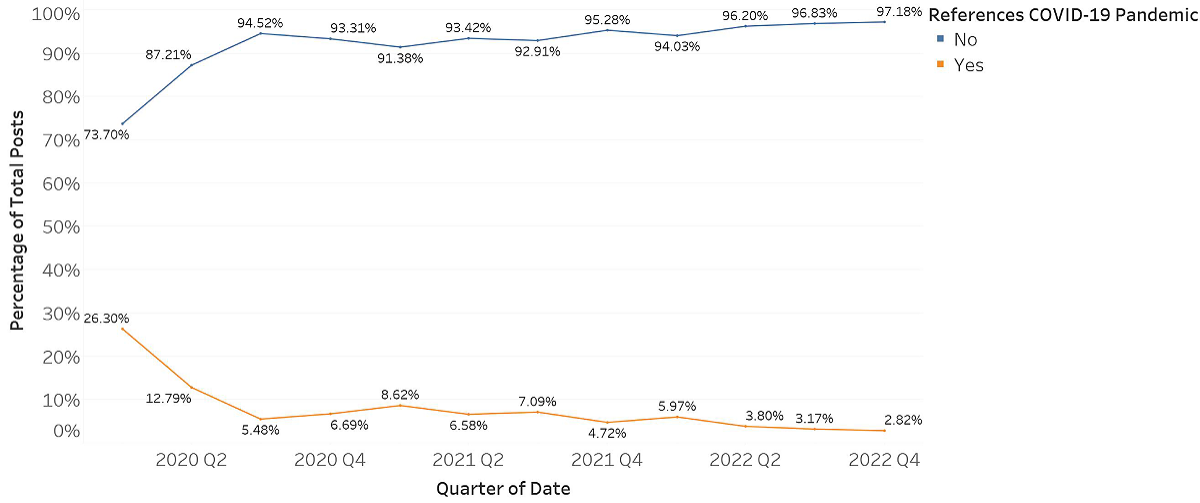
Percentage distribution of COVID-19 mentions throughout the study period.

During the study period, the model found that only a small portion of posts mentioned COVID-19 (n=3229, 6%) compared with those that did not (n=47,495, 89%). There were a small number of posts that were classified as unsure (n=2276, 4%). Although visual inspection of Figure 4 suggested a steep drop in COVID-19 mentions throughout the study period, chi-square results found a negligible difference in the number of references to COVID-19 (N=50,724; *χ*^2^_2_=460.21; *P*<.001; φ<0.001). Again, the investigation of effect sizes suggested these differences were negligible. Figures 2-4 were generated using Tableau Desktop [35]. An overview of the data is provided in Table 2.

**Table 2:** Data overview.

Characteristics		Final dataset
**Users**		
	Posts where author name was unknown, n	4013
	Posts by authors with single posts (ie, did not post more than once in the community), n	10,693
	Posts by authors with multiple posts (ie, more than one post in the community), n	38,627
	Posts per author, mean (SD)	2.6 (4.9)
**Posts, mean (SD)**		
	Length of title (characters)	48 (42)
	Length of body (characters)	590 (715)
**Engagement, mean (SD)**		
	Score	14 (37)
	Comments per post	10 (14)
**Communities, n**		
	r/CrohnsDisease	28,365
	r/UlcerativeColitis	20,394
	r/IBD	4574

## Discussion

### Principal Results

The main contributions of this study are threefold. First, using GPT-3.5, we implemented a novel approach to processing and categorizing social media discussions. Second, we assessed the model’s performance against human raters on a range of subjective and objective criteria. Third, we delved into the themes and emotions expressed by patients with IBD during the COVID-19 pandemic.

Our analysis of interrater reliability showcases that GPT-3.5, with prompt engineering, can achieve moderate interrater reliability on subjective aspects such as topic and emotions, and near-perfect reliability on objective elements such as age, gender, and COVID-19 mentions. Our successful use of this approach supports the preliminary feasibility of using GPT-3.5 and future iterations in analyzing big data.

Most posts did not disclose demographic information. However, among those who did, the overall demographics aligned with general Reddit usage. A notable observation was the presence of a small cohort of self-reported adolescents, highlighting a potential area for further investigation into pediatric patient discourse. Exploring the specific issues and experiences shared by this demographic can inform the development of tailored support mechanisms and educational materials that better address the needs of young patients with IBD and their families.

Most posts analyzed were straightforward questions or statements with neutral sentiment (n=43,896, 82%). For posts that had a sentiment value assigned, no single category had more positive sentiment than negative sentiment. The phenomenon toward negative sentiment values in health-related Reddit posts is consistent with findings in Goel et al [10] and Maleki et al [36]. The category with the highest ratio of positive to negative posts was diet, with an almost one-to-one ratio. Analysis of diet posts tends to show that while many people have issues with diet, many other people report success with being able to eat certain foods and finding “trigger foods.” The category with the lowest positive-to-negative post ratio is symptoms, with the overall lowest number of positive posts and highest number of negative posts. These posts often expressed issues surrounding pain and frequent bathroom use, as well as a lack of response to treatment. This finding reflects previous work highlighting that many posters appear to use health care–related social media to seek educational resources about their experiences and find validation for their symptoms from an empathetic internet-based community [10].

Consistent with previous studies, most discussions centered around medications (n=14,909, 28%) and symptoms (n=14,939, 28%). However, our analysis uncovered two distinct areas diverging from past research: dietary discussions were infrequent (n=3947, 7%), potentially due to the strong link between symptoms and dietary choices, and diagnosis-related posts, which constituted a small but significant portion of the dataset. A manual review revealed that these posts predominantly originated from individuals lacking a confirmed IBD diagnosis who were seeking diagnostic advice based on their symptoms. This emerging trend, previously undocumented, is concerning as it suggests a reliance on nonprofessional advice for health guidance. These data may support the need for greater community education regarding IBD, alongside outreach from the health care community to support individuals seeking a diagnosis. Finally, we also observed a gradual decline in pandemic-related mentions over the study period. This aligns with trends observed in other patient groups and suggests factors such as information fatigue or adaptation to the pandemic [37]. The reduced focus on COVID-19 among the IBD community, despite their heightened risk, underscores the need for ongoing research into the challenges faced by this population during the pandemic era.

### Limitations

Our analysis was subject to several limitations. During our data analysis, we used the GPT-3.5 Turbo endpoint, the leading model publicly available at that time. However, since then, OpenAI has released the GPT-4 model, which has shown improvement in capturing nuanced semantic information, an area where the GPT-3.5 model showed difficulties [38]. Furthermore, OpenAI plans to allow the GPT-4 model to be fine-tuned using manually annotated data, enhancing its accuracy. Future studies could use these more advanced models to score data more accurately.

Another limitation of our analysis lies in the nature of transformer models, such as GPT-3.5, used in this study. While these models are powerful, they lack transparency in their internal decision-making processes, making it difficult to fully understand how outputs are generated from inputs. This opacity can obscure potential biases, errors, or unintended correlations within the data, which may influence results in ways that are not readily apparent.

Further limitations are that Reddit’s user base, which differs in demographics such as age, gender, location, education, income, and interests from other internet-based communities, may limit the generalizability of our findings to other platforms. Second, we assigned each post to a single topic and sentiment category, potentially simplifying posts with multiple topics or mixed sentiments. Finally, we relied on self-reported data for the poster’s gender and age, which cannot be verified.

### Conclusion

In this study, we used GPT-3.5, a powerful pretrained NLP model, to analyze the posts from 3 IBD subreddits during the COVID-19 pandemic. We demonstrated the preliminary feasibility of GPT-3.5 as a valuable sentiment and topic analysis tool capable of producing results with moderate to near-perfect reliability with human raters. Our study helps to fill the knowledge gap surrounding the discourse of individuals diagnosed with IBD, especially in the context of the pandemic. We discovered that people with IBD expressed more negative than positive emotions and that their primary areas of discussion surround medication and symptoms. These findings highlight the challenges and concerns that people with IBD faced throughout the pandemic and suggest the need for more targeted support and education for this population. Our study also provides a validated dataset of IBD posts that can be used for further training future NLP models and would also be valuable for subgroup analyses conducted by gastroenterology-focused research teams.

## Data Availability

The dataset generated and analyzed for this study is not publicly available due to privacy concerns but is available from the corresponding author on reasonable request with institutional review board approval.

## Abbreviations

DHHS: Department of Health and Human Services
FDA: Food and Drug Administration
IBD: inflammatory bowel disease
NLP: natural language processing
VADER: Valence Aware Dictionary for Sentiment Reasoning

## References

[1] Dahlhamer J, Zammitti E, Ward B, Wheaton A, Croft J (2016). Prevalence of Inflammatory Bowel Disease Among Adults Aged ≥18 Years - United States, 2015. MMWR Morb Mortal Wkly Rep.

[2] Burke K, Kochar B, Allegretti J, Winter R, Lochhead P, Khalili H, Colizzo F, Hamilton M, Chan W, Ananthakrishnan A (2021). Immunosuppressive therapy and risk of COVID-19 infection in patients with inflammatory bowel diseases. Inflamm Bowel Dis.

[3] Cai Z, Wang S, Li J (2021). Treatment of inflammatory bowel disease: a comprehensive review. Front Med (Lausanne).

[4] Peterson J, Chesbro G, Larson R, Larson D, Black C (2021). Short-term analysis (8 weeks) of social distancing and isolation on mental health and physical activity behavior during COVID-19. Front Psychol.

[5] Chen J, Geng J, Wang J, Wu Z, Fu T, Sun Y, Chen X, Wang X, Hesketh T (2022). Associations between inflammatory bowel disease, social isolation, and mortality: evidence from a longitudinal cohort study. Therap Adv Gastroenterol.

[6] Nass B, Dibbets P, Markus CR (2022). Impact of the COVID-19 pandemic on inflammatory bowel disease: The role of emotional stress and social isolation. Stress Health.

[7] Rubin D, Torres J, Dotan I, Xu LT, Modesto I, Woolcott J, Gardiner S, Sands B (2024). An insight into patients' perspectives of ulcerative colitis flares via analysis of online public forum posts. Inflamm Bowel Dis.

[8] Rohde J, Sibley A, Noar S (2021). Topics analysis of Reddit and Twitter posts discussing inflammatory bowel disease and distress from 2017 to 2019. Crohns Colitis 360.

[9] Stemmer M, Parmet Y, Ravid G (2023). What are IBD patients talking about on Twitter? Using natural language understanding to investigate patients' tweets. SN Comput Sci.

[10] Goel R, Modhukur V, Täär K, Salumets A, Sharma R, Peters M (2023). Users' concerns about endometriosis on social media: sentiment analysis and topic modeling study. J Med Internet Res.

[11] Brown T, Mann B, Ryder N, Subbiah M, Kaplan J, Dhariwal P, Neelakantan A, Shyam P, Sastry G, Askell A, Agarwal S, Herbert-Voss A, Krueger G, Henighan T, Child R, Ramesh A, Ziegler D, Wu J, Winter C, Hesse C, Chen M, Sigler E, Litwin M, Gray S, Chess B, Clark J, Berner C, McCandlish S, Radford A, Sutskever I, Amodei D (2020). Language models are few-shot learners. Adv Neural Inf Processing Syst.

[12] Gao L, Biderman S, Black S, Golding L, Hoppe T, Foster C, Phang J, He H, Thite A, Nabeshima N, Presser S, Leahy C (2020). The pile: an 800GB dataset of diverse text for language modeling. ArXiv.

[13] Elyoseph Z, Hadar-Shoval D, Asraf K, Lvovsky M (2023). ChatGPT outperforms humans in emotional awareness evaluations. Front Psychol.

[14] Hackl V, Müller A, Granitzer M, Sailer M (2023). Is GPT-4 a reliable rater? Evaluating consistency in GPT-4's text ratings. Front Educ.

[15] Lamichhane B (2023). Evaluation of ChatGPT for NLP-based mental health applications. ArXiv.

[16] Wake N, Kanehira A, Sasabuchi K, Takamatsu J, Ikeuchi K (2022). Bias in emotion recognition with ChatGPT. ArXiv.

[17] Nadi F, Naghavipour H, Mehmood T, Azman AB, Nagantheran JAP, Ting KSK, Nor Adnan NMIB, Sivarajan R, Veerah S, Rahmat R, Wah YB, Al-Jumeily D, Berry MW (2024). Sentiment analysis using large language models: a case study of GPT-3.5. Data Science and Emerging Technologies: Proceedings of DaSET 2023.

[18] He L, Omranian S, McRoy S, Zheng K Using large language models for sentiment analysis of health-related social media data: empirical evaluation and practical tips. medRxiv.

[19] Lockwood A, Newman D, Mossing K, Glubzinski A, Cohen E Human vs. machine: a comparative analysis of qualitative coding by humans and ChatGPT-4. PsyArXiv.

[20] Reddit by the numbers. Reddit Inc.

[21] Cucinotta D, Vanelli M (2020). WHO declares COVID-19 a pandemic. Acta Biomed.

[22] Baumgartner J, Zannettou S, Keegan B, Squire M, Blackburn J (2020). The pushshift Reddit dataset. Vol. 14 (2020): Fourteenth International AAAI Conference on Web and Social Media.

[23] Watchful1 PushshiftDumps. GitHub.

[24] Alteryx.

[25] McHugh M (2012). Interrater reliability: the kappa statistic. Biochem Med (Zagreb).

[26] Hartling L, Hamm M, Milne A, Vandermeer B, Santaguida PL, Ansari M, Tsertsvadze A, Hempel S, Shekelle P, Dryden DM (2012). Validity and Inter-Rater Reliability Testing of Quality Assessment Instruments [Internet].

[27] Wongpakaran N, Wongpakaran T, Wedding D, Gwet K (2013). A comparison of Cohen's Kappa and Gwet's AC1 when calculating inter-rater reliability coefficients: a study conducted with personality disorder samples. BMC Med Res Methodol.

[28] Israel Glenn (1992). Determining Sample Size. University of Florida Cooperative Extension Service, Institute of Food and Agriculture Sciences.

[29] Watson Paul, Petrie Aviva (2010). Method agreement analysis: A review of correct methodology. Theriogenology.

[30] Yamane T (1967). Statistics: An Introductory Analysis.

[31] Bujang Mohamad, Baharum Nurakmal (2017). Guidelines of the minimum sample size requirements for Cohen’s Kappa. Epidemiology Biostatistics and Public Health.

[32] Gwet Kilem (2019). irrCAC: computing chance-corrected agreement coefficients (CAC). The Comprehensive R Archive Network.

[33] RStudio Team (2020). RStudio. Posit.

[34] (2024). Social media fact sheet. Pew Research Center.

[35] Tableau Desktop. Tableau.

[36] Maleki N, Padmanabhan B, Dutta K (2023). The emffect of monetary incentives on health care social media content: study based on topic modeling and sentiment analysis. J Med Internet Res.

[37] Zhang X, Yang Q, Albaradei S, Lyu X, Alamro H, Salhi A, Ma C, Alshehri M, Jaber I, Tifratene F, Wang W, Gojobori T, Duarte C, Gao X (2021). Rise and fall of the global conversation and shifting sentiments during the COVID-19 pandemic. Humanities and Social Sciences Communications.

[38] Achiam J, Adler S, Agarwal S, Ahmad L, Akkaya I, OpenAI (2024). GPT-4 technical report. ArXiv.

